# Dissemination of the Transmissible Quinolone-Resistance Gene *qnrS1* by IncX Plasmids in Nigeria

**DOI:** 10.1371/journal.pone.0110279

**Published:** 2014-10-23

**Authors:** Eric T. Sumrall, Elizabeth B. Gallo, Aaron Oladipo Aboderin, Adebayo Lamikanra, Iruka N. Okeke

**Affiliations:** 1 Department of Biology, Haverford College, Haverford, Pennsylvania, United States of America; 2 Department of Medical Microbiology and Parasitology, Obafemi Awolowo University, Ile-Ife, Osun State, Nigeria; 3 Department of Pharmaceutics, Obafemi Awolowo University, Ile-Ife, Osun State, Nigeria; U. S. Salinity Lab, United States of America

## Abstract

The plasmid-encoded quinolone resistance gene *qnrS1* was recently found to be commonly associated with ciprofloxacin resistance in Nigeria. We mapped the *qnrS1* gene from an *Escherichia coli* isolate obtained in Nigeria to a 43.5 Kb IncX2 plasmid. The plasmid, pEBG1, was sufficient to confer ciprofloxacin non-susceptibility, as well as tetracycline and trimethoprim resistance, on *E. coli* K-12. Deletion analysis confirmed that *qnrS1* accounted for all the ciprofloxacin non-suceptibility conferred by pEBG1 and tetracycline and trimethoprim resistance could be attributed to *tetAR* and *dfrA14* genes respectively. While it contained a complete IncX conjugation system, pEBG1 was not self-transmissible likely due to an IS*3* element inserted between the *pilX5* and *pilX6* genes. The plasmid was however efficiently mobilizable. pEBG1 was most similar to another *qnrS1*-bearing IncX2 plasmid from Nigeria, but both plasmids acquired *qnrS1* independently and differ in their content of other resistance genes. Screening *qnrS1*–positive isolates from other individuals in Nigeria revealed that they carried neither pEBG1 nor pNGX2-QnrS1 but that IncX plasmids were prevalent. This study demonstrates that the IncX backbone is a flexible platform that has contributed to *qnrS1* dissemination in Nigeria.

## Introduction

When antibacterial quinolones were first introduced into clinical practice, it was thought that resistance would be slow to appear and that transmissible resistance was improbable [1]. Initial reports of quinolone resistance were due to point mutations in the genes encoding their gyrase and topoisomerase targets that made them less sensitive to the drug. In 1998, Martinez-Martinez et al [2] described a plasmid-borne gene, now termed *qnrA*, which conferred four-to-sixteen-fold resistance to quinolones on Enterobacteriaceae. *qnrA* is a pentapeptide repeat protein that protects DNA gyrase from quinolone binding and inhibition [3]. Other *qnr* genes have since been reported and many can be transmitted horizontally. Transmissible quinolone resistance is also attributable to genes encoding plasmid-encoded efflux pumps, such as *qepA* and *oqx* and, in the case of ciprofloxacin, the acetylating enzyme *aac(6′)-Ib-cr*. While plasmid-encoded quinolone-resistance genes generally confer low-level resistance, their overall impact is great because they shield otherwise susceptible bacteria from the lethal effects of the quinolones, allowing them greater time and opportunity to evolve higher-level resistance.

Until recently, reports of quinolone resistance were almost non-existent from West Africa [4]. However, in the past decade Nigeria has seen a very rapid increase in fluoroquinolone use, due to the recent expiration of patents protecting ciprofloxacin and perfloxacin. Introduction of ciprofloxacin into Nigerian clinics was temporally associated with a significant rise in resistance among gut commensals. Five years after fluoroquinolones were introduced in a community in Western Nigeria, *Escherichia coli* strains showing quinolone-specific resistance mechanisms were isolated [5]. Although the majority of these isolates carried point-mutations in the quinolone-resistance determining regions of *gyrA* and *parC*, six strains bore the plasmid-encoded resistance gene *qnrS1*. In this study, we characterized a mobile element from one of these isolates in order to understand the mode of *qnrS1* dissemination in Western Nigeria.

## Methods

### Strains

Strain 09/22a and other *qnrS1*-bearing *E. coli* strains used in this study were isolated in 2009 during an earlier survey of quinolone resistance [5]. Other strains used in this study are listed in Table 1. Strains were maintained at −70°C in Luria broth: glycerol 1∶1.

**Table 1:** Strains used in this study.

	Genotype and description	Reference or Source
09/22a	*qnrS1*-positive *E. coli* isolate from Nigeria	[5]
DH5α	F^–^ ø80d*lacZ*ΔM15 Δ(*lacZYA-argF*)U169 *deo*R *recA*1 *endA*1 *hsdR*17(rK^–^ mK^+^) *phoA supE*44 λ– *thi*-1 *gyrA*96 *relA*1	Invitrogen
TOP10	F- *mcr*A (*mrr-hsd*RMS-*mcr*BC) ö80*lac*ZΔM15.*lac*X74 *deo*R *rec*A1 *ara*D139 *Δ*(*ara-leu*)7697 *gal*U *gal*K *rps*L (StrR) *end*A1 *nup*G	Invitrogen
C600 Nal^R^	Nalidixic acid-resistant derivative of C600.	[47]
SM10 λpir	λpir *E. coli* strain harboring an inc-P conjugation system in the chromosome	[35]
UMN026	Uropathogenic *E. coli* strain bearing IncP and IncX1 plasmids	[36]
NCTC 10418	Susceptibility testing control	
ATCC 35218	Susceptibility testing control	
LMG194	F-.*lac*X74 *gal* E *thi rps*L.*pho*A (*Pvu* II).*ara*714 *leu*::T*n1*0	Invitrogen [48]
EC1502	Rifampicin resistant *E. coli* strain	University of Bradford
J53	F^−^ *met pro* Azi^r^	[49]

### General molecular biology procedures

Genomic DNA was extracted using the Promega Wizard kit. Small-scale extractions of large, naturally occurring plasmids were carried out by a modified boiling protocol as described previously [6]. A Qiagen miniprep kit was used to extract smaller recombinant plasmids. Large scale preparations of plasmids over 20 Kb in size were prepared after growth in terrific broth and induction with chloramphenicol [7] using the Qiagen large construct kit. Plasmids were electroporated into *E. coli* host strains using a Biorad micropulser according to manufacturers' instructions. DNA amplification was performed using Platinum PCR Supermix (Invitrogen) and 1 µM oligonucleotide primer in each reaction. Oligonucleotide primer sequences are listed in Table 2. All amplifications began with a two-minute hot start at 94°C followed by 30 cycles of denaturing at 94°C for 30 s, annealing at 5°C below primer annealing temperature for 30 s, and extending at 72°C for one minute per kilobase of DNA. When the target PCR product was over 3 Kb, we used *Pfx* polymerase (Invitrogen) in accordance with manufacturer's instructions, with annealing at 5°C below the temperature used for *Taq* PCRs. Where necessary, for sequencing, PCR amplicons were TA cloned into the pGEM-T Easy vector (Promega) according to manufacturer's directions and plasmids were transformed into chemically competent *E. coli* K-12 TOP10 cells. For expression, amplicons were cloned into pBAD/Thio-TOPO (Invitrogen) and induced with 0.2% arabinose. Expression was verified by western blotting. All plasmids used or constructed in the course of this study are listed in Table 3. Other molecular biology operations were performed using standard procedures [8].

**Table 2:** Oligonucleotide primers used for PCR.

Target gene	Sequences	Application	Reference
topB	F 5' AGACTCTTTATTGCTGAAAAACCATC 3'; R 5' TTTTTTAGCGAATTCAAAACCTATTTTT 3'	Amplification of *topB* from pEBG1 for cloning into pBAD/Thio-TOPO	This study
fliC	F 5' ATGGCACAAGTCATTAATACCCAAC 3'; R5' CTAACCCTGCAGCAGAGACA 3'	*fliC* PCR-RFLP	[17]
qnrS	F 5' CAATCATACATATCGGCACC 3'; R 5' TCAGGATAAACAACAATACCC 3'	Amplification of *qnrS1*	[28]
IncX1 (taxC)	F 5' GCTTAGACTTTGTTTTATCGTT 3'; R 5' TAATGATCCTCAGCATGTGAT 3'	Amplification of *taxC* from IncX1 plasmids	[20]
IncX2 (taxC)	F 5' GCGAAGAAATCAAAGAAGCTA 3'; R 5' TGTTGAATGCCGTTCTTGTCCAG 3'	Amplification of *taxC* from IncX1 plasmids	[20]
qnrS1comp	F 5′ TGAGGGGTTGTAATGTGTTGAT 3′; R 5′ TGCAAGGTTGACAATATTATTCGTTTT 3′	Cloning *qnrS1*	This study
BlpIFrag	F 5′ AGTTGTTCCTCATGAG 3′; R 5′ ACTGACTGTCTTTGTATCA 3′	Verifying *qnrS1* deletion	This study
BlpI-2	F 5′ ATTCGCAGCGACTTTCGACGT 3′; R 5′TTCCGGTATCCGGGCTTCGGGTG 3′	Verifying *qnrS1* deletion	This study
pilX2	F 5′ ACCCTGTCTGTCTTCATGGC 3′; R 5′ ACCAGTTCGGCAGATAAGGC 3′	Amplification of *pilX2* from IncX plasmids	This study
pilX8	F 5′ GCACTTAATGCCGCTCTTCC 3′; F 5′ CCTGTACTGTTGCCGTTTGC 3′	Amplification of *pilX8* from IncX plasmids	This study

**Table 3:** Plasmids used in this study.

Plasmid	Description	Size	Reference or source
pEBG1	WT *qnrS1*-containing plasmid derived from *E. coli* strain 09/22a, confers resistance to ciprofloxacin, tetracycline and trimethoprim	43,530bp	This study
pETS4	pEBG1 plasmid with excised region containing *qnrS1*	41,387	This study
pINK2002	*qnrS1* cloned into the Tet gene of pACYC184	5,038 bp	This study
pETStop3	Expression vector (pBAD/Thio-TOPO vector) containing a *topB* insert	6,773 bp	This study
pBAD/Thio-TOPO	Arabinose inducible expression vector	4,454 bp	Invitrogen
p1ECUMN	IncP plasmid derived from *E. coli* strain UMN026	122,302 bp	[36]
p2ECUMN	IncX1 plasmid derived from *E. coli* strain UMN026	33,809 bp	[36]
pMG306	Naturally occurring *qnrS1*-bearing plasmid from a *Salmonella* isolate		[32]
pMB2	Naturally occurring *aac(6′)-Ib-cr*-bearing plasmid	125 Kb	This study
pMB80-2	Naturally occurring conjugative plasmid from enteropathogenic *E. coli* strain	>100 Kb	[34]
pGEM-T	Amp^R^; TA-cloning vector	3,000 bp	Promega
pACYC184	Tet and Cm resistant cloning vector	4,244 bp	NEB

### Shot-gun sequencing and sequence analysis

Whole-replicon shotgun library preparation, Sanger sequencing and assembly of a large plasmid were performed by SeqWright DNA Technology Services (Houston, TX). Sequence analyses and annotation were performed in Artemis [9]. Open reading frames were initially defined by Glimmer. Annotations were made where BLAST e-values equaled or approached zero and there was 98% or greater identity at the nucleotide and amino acid levels. Direct and inverted repeats were identified by dot-plot analysis of pairwise FASTA alignments made using the BLAST suite. Open reading frame (Orf) and feature plots were prepared using Artemis and DNAPlotter [10]. Orf identify was determined using BLAST and Pfam [11], [12]. Multiple sequence alignments were performed using Clustal and dendograms were computed using a Jones-Taylor-Thornton (JTT) model in MEGA5 [13].

### Antimicrobial susceptibility testing

Antimicrobial susceptibility testing by disc diffusion was performed using the Clinical and Laboratory Standards Institute (CLSI, formerly NCCLS) [14]. Antimicrobial discs and control strain *E. coli* ATCC 35218 were obtained from Remel. The antimicrobial discs used contained ampicillin (10 µg), streptomycin (10 µg), trimethoprim (5 µg), tetracycline (30 µg), nalidixic acid (30 µg), chloramphenicol (30 µg), ciprofloxacin (5 µg) and sulphonamide (300 µg). Inhibition zone diameters were interpreted in accordance with CLSI guidelines with WHONET software version 5.3 [15]. Minimum inhibitory concentrations (MICs) to nalidixic acid were measured by the E-test (Biomerieux), in accordance with manufacturer's instructions and by the broth dilution technique in Mueller-Hinton broth as recommended by the CLSI and using *E. coli* ATCC 35218 as control [16].

### *In vitro* conjugation

*In vitro* conjugation experiments were performed by solid and liquid mating. Donors and recipients were cultured in LB with appropriate selective antimicrobials. For liquid mating, 50 µl and 200 µl of mid-logarithmic phase LB cultures from donor and recipient respectively were mixed in 1 mL of LB, pre-warmed to 37°C. After one hour mating at 37°C, the cells were placed on ice to terminate the reaction. For solid mating, 0.5 mL of donor and recipient culture, grown overnight with selection, was spun down gently and resuspended in 20 µl of LB without antibiotics. The suspension was spotted onto dried LB plates, allowed to dry at room temperature for 15 minutes and then incubated at 37°C for three hours or overnight. The mating reaction was resuspended in 1 ml of LB with vortexing and placed on ice to terminate conjugation. After mating, serial ten-fold dilutions of each terminated reaction was made in cold phosphate buffered saline and plated onto plates containing tetracycline (or other appropriate antimicrobials for controls) to select for the plasmid and nalidixic acid, resistance to which is conferred chromosomally in the recipient. Transconjugant colonies were counted after overnight incubation at 37°C and verified by plasmid profiling, phenotype on MacConkey and Eosin methylene blue agars, PCR-RFLP for the *fliC* allele [17], and PCR for donor- and recipient-specific markers. Viable counts of donors and recipient were also performed. The number of transconjugant colonies per donor colony-forming units was computed as the plasmid transfer efficiency [18].

### Plasmid stability

We serially passaged pEBG1 in its source strain, 09/22a and in DH5αE essentially as described by Sandegren et al [19]. Triplicate starting cultures were grown overnight at 37°C in 1 mL of LB supplemented with tetracycline (25 mg/L). Bacterial cells were washed by spinning down overnight cultures and resuspending the pellet in 1 mL of LB without antibiotics. An aliquot of 100 µL of washed cells was transferred into 1 mL of LB and incubated overnight at 37°C. Serial passaging of 100 µL of overnight culture to 1 mL of LB was performed daily, approximating 10 generations of growth per passage. Every 20 generations (48 hours), samples were diluted and plated on MacConkey and tetracycline plates. Once counts began to drop off, colonies were screened through replica plating on plates containing tetracycline, trimethoprim and ciprofloxacin to confirm that cells lost the plasmid.

## Results

### Quinolone-non-susceptible strain 09/22a from Nigeria carries its *qnrS1* gene on an IncX plasmid

**Figure 1:**
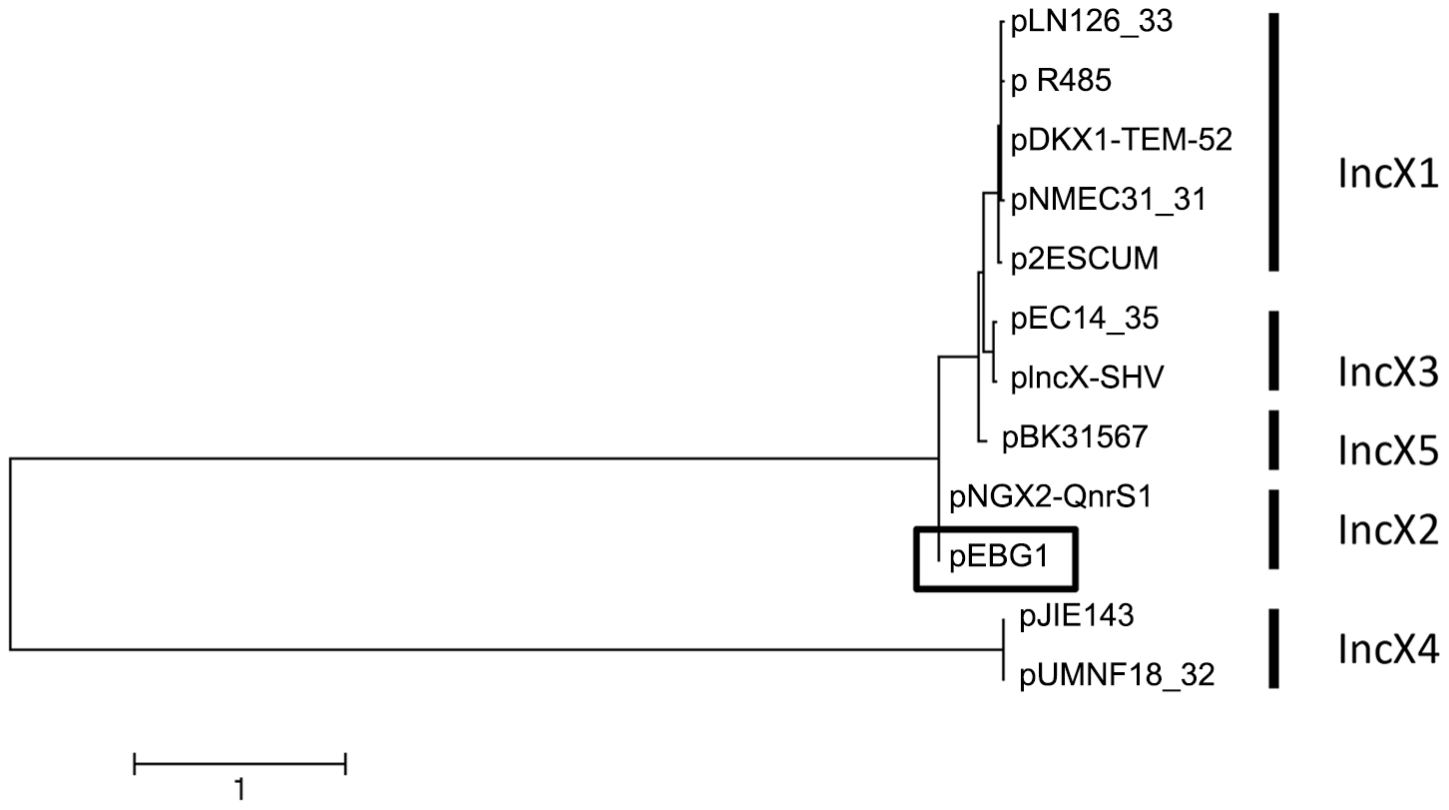
a. Maximum likelihood dendogram depicting evolutionary relationships among TaxC sequences from 12 IncX plasmids, including representatives from groups defined from whole plasmid polymorphisms [20] and pEBG1(boxed), the plasmid sequenced in this study.

Strain 09/22a is a quinolone-non-susceptible *E. coli* isolate from Nigeria that tested positive for *qnrS1* by PCR in a recent study [5]. The strain was unable to conjugate quinolone resistance to *E. coli* C600, TOP10 or EC1502. We therefore extracted plasmids from 09/22a by boiling and electroporated the extract into electrocompetent *E. coli* DH5αE (Invitrogen). Selection on nalidixic acid plates (100 µg/mL) yielded no transformants. Selection on plates containing 1 mg/mL of ciprofloxacin produced transformants resistant to ciprofloxacin, trimethoprim and tetracycline, all of which carried a single large plasmid. The plasmid, termed pEBG1, was purified and shot-gun sequenced. pEBG1 was found to be to be 43,534 bp, with an overall G+C content of 46.72%. Its sequence has been deposited in Genbank (Accession number KF738053). pEBG1 is most similar to IncX2 plasmid pNGX2-QnrS1(Genbank accession no. JQ269335) [20] and the two plasmids have 100% identical *taxC* genes. (IncX plasmids are sub-classified based on TaxC, which agrees with subclassifications based on polymorphic sites across whole plasmid sequences [20]). All other *taxC* genes are less than 90% identical to those of pEBG1 and pNGX2-QnrS1 and therefore pEBG1 is an IncX2 plasmid (Figure 1). The IncX2 plasmids demonstrate significant homology to other IncX plasmids, including the prototype R6K, with the most similarity occurring in the conjugative pilus operon [21].

**Figure 2:**
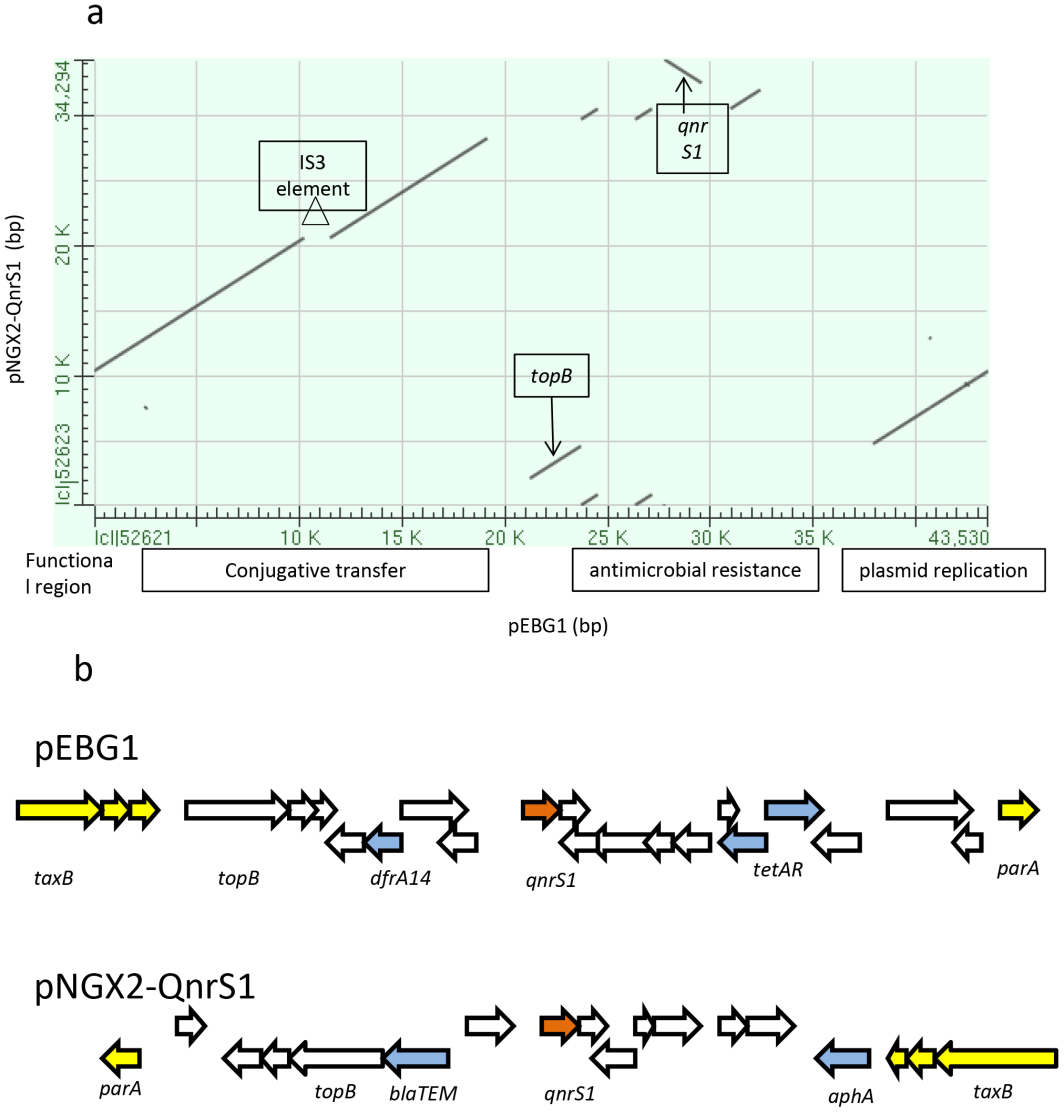
a. Dot plot of pairwise alignment between incX2 plasmids pEBG1 on the x-axis and pNGX2-QnrS1 on the y-axis. The plasmids are highly conserved overall but differ in the location and content of their antimicrobial resistance modules. The pEBG1 conjugation system is interrupted by an IS*3* element not present in pNGX2-QnrS1. b. schematic of the resistance regiosn of plsdmifd pEBG1 and pNGX2-QnrS1. *qnrS1* is colored brown and other antimicrobial resistance genes are shaded blue. The flanking core plasmid regions are colored yellow. Note that the insertions are in diffirent orientations.

As was pEBG1, pNGX2-QnrS1 was isolated in western Nigeria (Genbank accession no. JQ269335) [20] and the two plasmids currently represent the only completely sequenced IncX2 plasmids in the database. 71% of pEBG1 is 99% identical to 90% of pNGX2-QnrS1 and both plasmids carry the *qnrS1* gene (Figure 2). The backbones, including the region encoding the conjugation system, are nearly identical between the two plasmids, strongly suggesting that they share a recent ancestor. Their resistance modules are integrated at the same site butas shown in Figure 2, the plasmids diverge in the predicted mobile elements and resistance genes they carry and their resistance modules, based on the orientation of the *qnrS1* gene, are inserted in opposite orientations.

**Figure 3:**
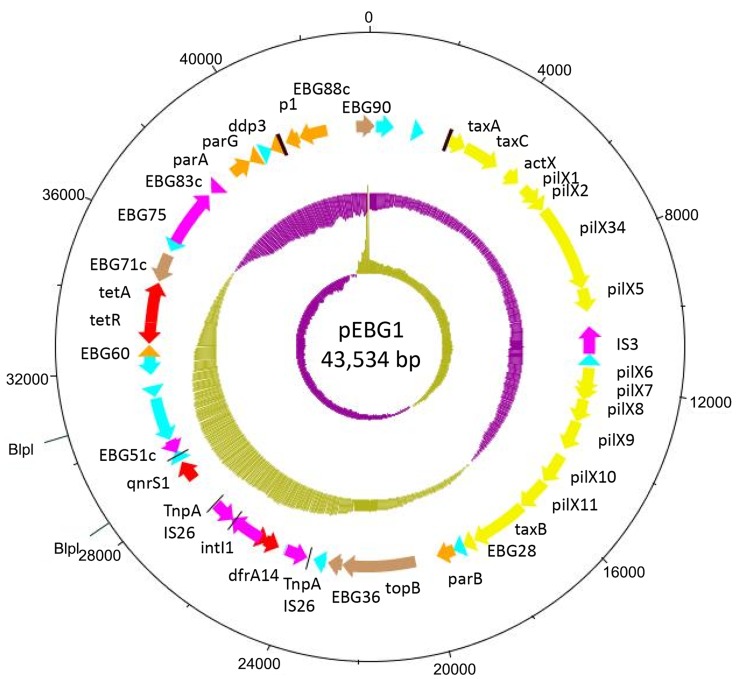
Circular map of pEBG1. and BlpI restriction sites conjugation genes are yellow and those that are predicted to encode proteins involved in DNA mobility or transposition are pink. Orange colored genes are involved in plasmid maintenance; other predicted open reading frames colored brown and hypothetical open-reading frames are colored light blue. Predicted OriT sites are marked as heavy dark brown lines. Thin black lines indicate the positions of IS*26* repeats.

Like other IncX plasmids, pEBG1 contains alpha and beta origins of replication, and carries the necessary genes to facilitate plasmid replication and partition: *p1*, a replication initiation protein, *ddp3*, a DNA distortion polypeptide which alters the topology of the DNA to allow for transcription initiation (similar to *taxA* and *taxC*), and *parA* and *parG* which are plasmid partition proteins involved in segregation and stability [21]. As shown in Figure 3, the pEBG1 plasmid backbone contains all other core genes common to IncX plasmids, in the following order: *taxA*, encoding a DNA-distortion polypeptide, which is necessary for altering the DNA helix of plasmids that contain two replication origins [22]; *taxC*, encoding a relaxase required for replication and conjugative transfer of the plasmid [21]; *actX*, containing a NusG transcription termination motif and probably the last gene in that operon; the IncX-type pilus synthesis operon (*pilX1*-*pilX11*); putative relaxase, *taxB*; and putative nuclease *parB*. pEBG1 contains an IS*3* element in its pilus synthesis operon between *pilX5* and *pilX6*. This insertion sequence is not present in pNGX2-QnrS1 (Figures 2a and 3).

pEBG1 also carries open reading frames (orfs) not found in all IncX plasmids, including *orfEBG036*, predicted to encode a H-NS histone family protein, EBG071c, predicted to encode a PecM-like membrane protein associated with the *tetAR* genes, and a putative DnaJ-domain protein that is 39% similar to the N-terminal J-Domain of the *E. coli* heat-shock chaperone DnaJ/Hsp40. DnaJ-domain proteins are often encoded on plasmids, including pNGX2-QnrS1, but their function in this context is unknown. The function of the putative ABC transporter permease (PecM)-like protein is also unknown. Proteins belonging to this family are involved in small molecule efflux. Plasmid-borne H-NS family proteins are known to compete with chromosomal H-NS, reducing repression of A-T rich sequences present on the plasmid [23]. (The region containing the 657 bp *qnrS1* orf and 570 bp of intergenic sequence upstream, conserved among *qnrS1*-bearing plasmids, has a GC% of 44.42%, compared to 46.72% for the whole plasmid and 50.8% for the *E. coli* chromosome [24]). Another gene found on pEBG1, which is also found in many, but not all, other IncX-type plasmids, is the 2.3 Kb *topB*, coding for bacterial type III topoisomerase. *topB* is truncated at the 5′ end in pNGX2-QnrS1 by an antimicrobial resistance gene module but pEBG1 contains a full-length gene. Type III topoisomerases decactenate DNA during replication [25] but the function of plasmid-encoded *topB* genes, which are only partially conserved, has not been elucidated.

pEBG1 confers resistance to trimethoprim, tetracycline and the quinolones. The plasmid's trimethoprim resistance gene, *dfrA14*, probably originated in a class 1 integron. The 5′ end of this integron, including the *intI*1 gene is intact. The integron also includes a truncated *aadA* gene. The 3′ end of the integron is truncated by an IS*26* element that is 99% identical to the element downstream of *qnrS1*. Although both carry *qnrS1*, the region containing the *dfrA14* gene is not present on the similar plasmid pNGX2-QnrS1, suggesting that it has been recently mobilized, perhaps via the IS*26* element. The resistance module encompassing *qnrS1* and *its* flanking resistance and transposase genes is nearly identical to that of other plasmids, including *E. coli* plasmid pT078 and pKOX105, from a *Klebsiella oxytoca* strain, with the important exception that beta-lactamase genes present on these plasmids are absent in pEBG1 [26], [27]. Although the *qnrS1* modules of pEBG1 and pNGX2-QnrS1 are identical, the plasmid backbones are also highly similar, and the two plasmids were recovered from geographically proximal areas, it is clear that *qnrS1* was acquired independently by each plasmid. The location of *qnrS1* and its flanking IS*26* element varies between the plasmids, being at the 5′ end of *topB* in pNGX2-QnrS1 but with the resistance region downstream of *topB* in pEBG1. *qnrS1* is often adjacent to a *tnpA* transposase in other plasmids, and it has been hypothesized that the *qnrS1* gene is usually part of an IS*26* element [28]. IS*26* contains two 14 bp perfect terminal repeats. We identified four IS*26*-associated inverted repeats [29] adjacent to the *qnrS1* gene. These 14 bp sequences begin at coordinates 23,698, 24,504, 27,142 and 29,074, marking out a putative transposable element of 5,376 bp encompassing *dfrA14* and *qnrS1*.

In addition to *qnrS1* and *dfrA14*, pEBG1 also contains the genes *tetR* and *tetA*, which lie adjacent to the *pecM*-like gene EBG71 and another *tnpA* gene in a high G+C content region, whose collective sequence was also not found on pNGX2-QnrS1 (Figure 2). These genes are bordered by a putative relaxase and Tn*3* transposase, which outline the boundaries of the elevated GC content area, suggesting that the plasmid's tetracycline resistance also recently came into the plasmid via a transposable element. The existence of this module on other resistance plasmids [30], and its absence from homologous plasmid pNGX2-QnrS1 support this idea. No other areas of the plasmid show a significant difference in G+C content, save for the fact that the backbone of the plasmid has an overall lower G+C content than the rest of the plasmid (Figure 3).

### *qnrS1* is the sole quinolone resistance-conferring gene on pEBG1

pEBG1 encodes a number of hypothetical ORFs as well as ORFs that could contribute to topoisomerase activity (TopB) or antimicrobial efflux (PecM-like EBG071c). To verify that *qnrS1* is the only quinolone resistance gene on pEBG1, we deleted the gene by restricting the plasmid with *Blp*I (which cuts at positions 28,514 and 30,657 in our annotation effectively removing 2,143 bp, which includes the 3′ half of *qnrS1* as well as two other hypothetical orfs, Figure 3). The resulting plasmid, pETS4 was confirmed by PCR analysis with the *qnrS1*F and R primer pair and restriction analysis with *Nru*I, which has 10 sites in pEBG1 including a site at position 30,368, within the deleted region. pETS4 conferred a six-fold lower MIC to ciprofloxacin than pEBG1 on DH5α and a greater than 32-fold lower MIC on TOP10. Resistance was restored upon trans-complementation with *qnrS1* clone pINK2002. Deleting *qnrS1* from pEBG1 however produced little effect (about 2–3 fold) on MICs to nalidixic acid. We then determined the level of resistance conferred by *aac(6′)-Ib-cr*, a gene that confers ciprofloxacin resistance but not resistance to other quinolones [31], using plasmid pMB2, which carries this gene and no other known plasmid-mediated quinolone resistance gene. We found that pMB2 provided no significant alteration to the nalidixic acid MIC of DH5α, but increased resistance to ciprofloxacin three-fold. As *qnrS1* has previously been reported to confer significant resistance to multiple quinolones, including nalidixic acid, we additionally tested pMG306, a *qnrS1*-bearing plasmid isolated from non-typhoidal *Salmonella* [32]. As in the original report describing the plasmid [32], we confirmed that this plasmid conferred only a slightly greater level of resistance on TOP10, compared to pEBG1, and that ciprofloxacin resistance was increased over 10-fold in contrast to four-fold increase in nalidixic acid resistance. Similar results were seen with this plasmid in the DH5α andJ53 backgrounds. Thus *qnrS1* principally confers ciprofloxacin resistance. In addition to demonstrating the pivotal role that *qnrS1* plays in ciprofloxacin resistance, these data demonstrate that the host background is an important determinant of the effect of *qnrS1* (Table 4).

**Table 4:** MICs to quinolones conferred by pEBG1, its derivatives and other Qnr-encoding plasmids.

Strain	Resistance to non-quinolone antimicrobials	Nalidixic acid MIC	Ciprofloxacin MIC
09/22a	Amp Chl Str Sul Tet Tmp	12	0.38
C600	-	>256	0.5
DH5α	-	24	0.032
DH5α (pEBG1)	Tet Tmp	12	0.125
DH5α (pETS4)	Tet Tmp	6	0.032
DH5α (pETS4, pINK2002)	Chl Tet Tmp	8	0.016
DH5α (pETS4, pACYC184)	Chl Tet Tmp	6	0.008
DH5α (pMG306)	Chl	24	0.25
DH5α (pMB2)	Tet Tmp	24	0.094
			
TOP10	Str	0.75	<0.002
TOP10 (pEBG1)	Str Tet Tmp	2	0.064
TOP10 (pETS4)	Str Tet Tmp	0.75	<0.002
TOP10 (pETS4, pINK2002)	Chl Str Tet Tmp	1	0.016
TOP10 (pETS4, pACYC184)	Chl Str Tet Tmp	0.75	<0.002
TOP10 (pMG306)	Chl	3	0.19
J53	Azi	4	0.008
J53 (pMG306)	Azi Chl	24	0.75
J53 (pMG282)	Azi Chl Str	16	0.38

*topB* is a large, partially conserved gene that is present in most IncX plasmids, but the function of plasmid-encoded topoisomerases is unknown. Chromosomally encoded DNA gyrase and topoisomerase are the normal targets of the quinolones. While *topB* genes, encoding type III topoisomerases, should not normally be inhibited by antibacterial quinolones, which are better inhibitors of type II topoisomerases, bacterial *topB* genes have not previously been evaluated in this regard. Since over-expression of chromosomal topoisomerases can compensate for absence of gyrase [25], it is possible that plasmid-encoded TopB could confer resistance to the quinolones by supplementing the activity of inhibited chromosomal topoisomerases. As it is possible that *topB* is not expressed under conditions we used to test for quinolone resistance *in vitro*, we cloned the gene and placed it under the control of the arabinose promoter, using the vector pBAD/Thio-TOPO (Invitrogen). The resulting clone, pETS-Topo3 produced copious amounts of the TopB protein when induced with 0.2% arabinose. Expression was abrogated in the presence of 2% glucose (data not shown). MICs of the clone to nalidixic acid and ciprofloxacin were identical in arabinose and glucose, confirming the *topB* is not sufficient to confer quinolone resistance. Because it is known some *topB* alleles can promote homologous recombination by stabilizing and resolving Holliday junctions [33], we hypothesized that *topB* might enhance mutation rates and that this might serve as a mechanism of evolution to antimicrobial resistance by strains carrying IncX plasmids like pEBG1. However, tests with the pETS-Top3 clone in a TOP10 and LMG194 background did not reveal enhanced mutation rates leading to resistance to rifampicin determined by plating cultures on LB plates containing 50 µg/mL rifampicin (data not shown).

### pEBG1 is mobilizable but not self-transmissible

We performed conjugation experiments on solid and in liquid media, using pMB80-2 as a control plasmid [34]. We were unable to conjugate pEBG1 into any of three recipient strains, C600, TOP10 or EC1502 even when mating was extended to 24 h. To determine whether pEBG1 could be mobilized *in vitro*, we electroporated it into strains carrying self-transmissible conjugation systems. Strain SM10, which contains conjugation genes from the broad host range IncP-type plasmid RP4 integrated into its chromosome [35] was unable to mobilize pEBG1. We then transferred two plasmids, IncP plasmid p1ECUMN and IncX1 plasmid p2ECUMN, from UMN026 [36], into C600 (Nal^R^) by conjugation. The presence of both plasmids in strain C600 was confirmed by plasmid profiling and PCR. C600 (p2ECUMN, p1ECUMN, pEBG1) could mobilize pEBG1 into TOP10 (Strep^R^). The conjugation rate for pEBG1 after a 3-hour mating was 1.7×10^−3^. At 24 hours it was 3.38×10^−7^.

### Other *qnrS1*-bearing isolates from Western Nigeria carry non-identical plasmids

We screened genomic DNA from other *qnrS1*-bearing isolates recovered in the same study for pEBG1 plasmids. All these strains show lowered susceptibility to ciprofloxacin but only one meets the CLSI breakpoint criteria for resistance. The strains were screened using *taxC*-based primers designed by Johnson et al (2012) [20], as well as primers specific for *qnrS1, topB, pilX2* and *pilX8*. As shown in Table 5, all five pEBG1 markers were detected in another strain isolated from the same individual but not in the other isolates. As we were able to obtain two or more of the pEBG1 marker amplicons from three other strains, we screened them for the IncX1 *taxC* gene and found that only two strains, including 09/22a carried IncX1 plasmids.

**Table 5:** IncX plasmid types in *qnrS1*-positive isolates from Nigeria.

Strain	Resistance pattern	*incX1*	*incX2*	*pilX2*	*pilX8*	*topB*	*qnrS1*
09/5d	Amp **Nal** Str Sul Tet Tmp	-	-	-	-	-	+
09/9a	Amp Chl **Nal** Str Sul Tet Tmp	-	-	-	-	-	+
09/16e	Amp Chl **Nal** Str Tet Tmp	-	-		+		+
09/22a	Amp Chl **Nal** Str Sul Tet Tmp	+	+	+	+	+	+
09/22d	Amp Chl **Nal** Str Tet Tmp	+	-	+	+	+	+
09/24c	Amp **Cip** Chl **Nal** Str Sul Tet Tmp	-	-	-	-	-	+
09/18d	Amp Chl **Nal** Str Sul Tet Tmp	-	+	-	-	-	+
UMN206		+	-	+	+	+	-
DH5α		-	-	-	-	-	-

### pEBG1 is propagated in the medium term without selection

**Figure 4:**
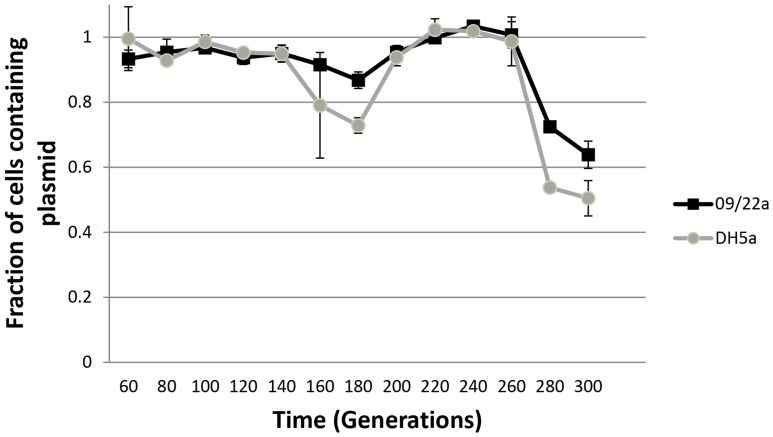
Stability of pEBG1 in the absence of selection in its natural host, 09/22a, and in laboratory strain DH5α.

We studied the stability of pEBG1 following serial passage. pEBG1 was stably inherited by its original host strain 09/22a and by DH5α for over 200 generations without selection (Figure 4). However, extended subculture lead to a significant proportion of those lines loosing the plasmid.

## Discussion

Plasmids harboring *qnrS* genes tend to be on average smaller than plasmids carrying other *qnr* subtypes, and are less likely to be self-transmissible [1], [37]. They vary greatly, belonging to N, L, M, H, R and X incompatibility groups and residing in diverse enterobacterial genera [22], [37]. Multiple studies have also noted that *qnrS1* tends not to be associated with integrons, and are instead associated with *tnpA* transposase genes, which likely contribute to intermolecular mobility [22], [28], [38].

In this study, we sequenced and functionally characterized a *qnrS1*-bearing plasmid obtained from an *E. coli* isolate in south-west Nigeria. At the time of isolation, *qnrS1* was the sole plasmid-encoded quinolone resistance gene identified in that locality and since the isolates were not clonal, we hypothesized that one or a few plasmids might account for the recently emerged quinolone resistance seen in the community. We identified and characterized pEBG1, a 43.5 Kb IncX2 plasmid. We find that other strains from Nigeria carry similar plasmids but that pEBG1 does not appear to have clonally expanded.

The IncX2 plasmid we characterized carries, in addition to *qnrS1*, genes conferring resistance to tetracycline and trimethoprim. The resistance gene repertoire is the most common variable among *qnrS1*-bearing IncX plasmids in and beyond Nigeria. This finding is worrisome, since it suggests that IncX backbones may be good scaffolds for incorporating new resistance genes. For example, in addition to plasmid-encoded quinolone resistance genes, extended-spectrum beta-lactamase genes have also been recently found on IncX plasmids [39], . Although the plasmid we identified in this study was from a human isolate, IncX plasmids, including one IncX2 plasmid, bearing *qnrS1* have also been found in isolates from migratory birds in Europe [41] and pNGX2-QnrS1 is a poultry isolate, pointing to possible animal reservoirs and global reach of these elements.

Qnr proteins confer low-level resistance by altering the interaction between the quinolones and their targets. What is known about Qnr function and spectrum has been gleaned from mechanistic studies with QnrA and QnrB. In this study, we found that *qnrS1* from pEBG1 conferred significant fluoroquinolone but only modest quinolone resistance on laboratory *E. coli* isolates. The finding that *qnrS1* confers ciprofloxacin non-susceptibility but very little nalidixic acid resistance on these strains is not wholly surprising since a previous study has indicated that *qnrS1* gene products sometimes confer no nalidixic acid resistance [42]. A separate study found that *qnrS* confers moderate nalidixic acid and ciprofloxacin resistance in *E. coli* strain HB101, but confers no resistance at all in *Shigella* strains [43]. The *qnrS1* gene was originally reported from a 47 Kb conjugative plasmid, pAH0376, from Japan in 2005 [43]. The plasmid was reported to confer on *E. coli* HB101 resistance to nalidixic acid (MIC, 16 µg/mL) and ciprofloxacin (MIC, 0.25 µg/mL). The following year, plasmid-borne *qnrS1* was reported from Germany and although the responsible plasmid pINF1 was not identical to pAH0376, the *qnrS1* genes on both plasmids were flanked by a Tn3 transposon carrying a *bla*_TEM-1_ gene. Other contexts for *qnrS1* have since been described [37], [41], [42]. Most of these reports demonstrate that *qnrS1* confers low- to intermediate resistance to ciprofloxacin (0.25–0.5 µg/mL). Altogether, the findings of this and earlier studies indicate that the nature and degree of quinolone resistance conferred by *qnrS1* is dependent on host strain background [42]. In Nigeria, most of the quinolone resistance selective pressure comes from ciprofloxacin, as in Vietnam [37], where *qnrS1* is also highly prevalent, and it therefore makes sense that genes preferentially conferring resistance to this agent will be selected.

This research, along with work from other laboratories [20], [44], points to multiple instances of *qnrS1*-bearing IncX plasmids in Nigeria. In this study, we determined that plasmid pEBG1 is highly mobilizable, though not self transmissible. The model IncX plasmid R6K is reported to conjugate at a rate of 1.7×10^−3^ into *E. coli* strain MC1061 [45], the same as the mobilization rate for pEBG1. The most likely explanation for the inability of pEBG1 to mediate its own conjugation is the insertion of an IS*3* element between *pilX5* and *pilX6*. Although this element does not disrupt a gene, it does interrupt the *pilX* operon and is likely polar on downstream genes. As the IS*3* element is absent from pNGX2-QnrS1, we anticipate that self transmissible IncX plasmids are also in circulation in Nigeria. Resistant strains in Nigeria frequently carry multiple plasmids [46]. Therefore the lack of a fully functional conjugative system is unlikely to greatly compromise the success of this *qnrS1*-bearing plasmid as long as it remains mobilizable. Overall, the high transmission rate and modular content observed by us and others [20], [45] illustrates that IncX plasmids are good candidates for mediating the dissemination of resistance genes. pEBG1 was stably inherited without selection in the medium term but was lost after 16–18 passages. This suggests that IncX2 plasmids bearing *qnrS1* may be maintained by antibacterial drug selection and therefore their frequent occurrence in Nigeria may be linked to overuse of antibacterial drugs there, among which ciprofloxacin, trimethoprim and tetracycline are prominent.
